# The Mechanisms of Cucurbitacin E as a Neuroprotective and Memory-Enhancing Agent in a Cerebral Hypoperfusion Rat Model: Attenuation of Oxidative Stress, Inflammation, and Excitotoxicity

**DOI:** 10.3389/fphar.2021.794933

**Published:** 2021-12-10

**Authors:** Zhiyong Liu, Manish Kumar, Sushma Devi, Atul Kabra

**Affiliations:** ^1^ Henan University of Traditional Chinese Medicine, Zhengzhou, China; ^2^ Chitkara College of Pharmacy, Chitkara University, Punjab, India; ^3^ Department of Pharmacy, Guru Nanak Institute of Technology, Ambala, India; ^4^ University Institute of Pharma Sciences, Chandigarh University, Mohali, India

**Keywords:** cerebral hypoperfusion, cucurbitacin E, memory, GABA, bay-K8644, caspase-3, inflammation, working memory

## Abstract

Impaired cerebral hemodynamic autoregulation, vasoconstriction, and cardiovascular and metabolic dysfunctions cause cerebral hypoperfusion (CH) that triggers pro-oxidative and inflammatory events. The sequences linked to ion-channelopathies and calcium and glutamatergic excitotoxicity mechanisms resulting in widespread brain damage and neurobehavioral deficits, including memory, neurological, and sensorimotor functions. The vasodilatory, anti-inflammatory, and antioxidant activities of cucurbitacin E (CuE) can alleviate CH-induced neurobehavioral impairments. In the present study, the neuroprotective effects of CuE were explored in a rat model of CH. Wistar rats were subjected to permanent bilateral common carotid artery occlusion to induce CH on day 1 and administered CuE (0.25, 0.5 mg/kg) and/or Bay-K8644 (calcium agonist, 0.5 mg/kg) for 28 days. CH caused impairment of neurological, sensorimotor, and memory functions that were ameliorated by CuE. CuE attenuated CH-triggered lipid peroxidation, 8-hydroxy-2′-deoxyguanosine, protein carbonyls, tumor necrosis factor-*α*, nuclear factor-kappaB, myeloperoxidase activity, inducible nitric oxide synthase, and matrix metalloproteinase-9 levels in brain resulting in a decrease in cell death biomarkers (lactate dehydrogenase and caspase-3). CuE decreased acetylcholinesterase activity, glutamate, and increased *γ*-aminobutyric acid levels in the brain. An increase in brain antioxidants was observed in CuE-treated rats subjected to CH. CuE has the potential to alleviate pathogenesis of CH and protect neurological, sensorimotor, and memory functions against CH.

## Introduction

Cerebral hypoperfusion (CH) originates from cardiovascular abnormalities that result in severe neurobehavioral deficits similar to Alzheimer’s-type dementia and vascular cognitive impairment and dementia (Dong et al., 2018). A decease in cerebral blood flow (CBF) is typically observed in old age along with other comorbidities that share some major risk factors, such as cardiac disorders (e.g., cardiac arrest), hypertension, atherosclerosis, dyslipidemia, and metabolic diseases (e.g., hyperglycemia, obesity) (Duncombe et al., 2017). The brain is vulnerable to hemodynamic alterations owing to the need of an uninterrupted supply of glucose and oxygen to meet the energy requirements for fulfillment of metabolic demands. However, vascular impairments and deregulation of hemodynamic autoregulatory mechanisms cause hypoperfused states in the brain that give rise to a sequence of events detrimental to brain architecture and functions (Liu and Zhang, 2012; Saxena et al., 2015). At present, the pharmacotherapeutic approaches in patients of CH are limited to symptomatic treatment only, and there is a dearth of some novel therapeutic agents that can modify or even reverse the pathogenesis of CH (Farkas et al., 2002).

Hemodynamic aberrations hamper aerobic respiration and energy supply that results in failure of ion-channel activity and biogenesis of reactive oxygen species in the mitochondria. A pathogenic increase in free radicals not only depletes endogenous antioxidants, but also instigates catastrophic events of oxidative stress and inflammation in the hypoperfused brain (Chen et al., 2011). Ion channelopathy, such as hyperactivation of postsynaptic *N*-methyl D-aspartate receptors (NMDAR), leads to a calcium (Ca^2+^) influx that initiates proteolytic mechanisms (e.g., calpains) and Ca^2+^-dependent cell death pathways (e.g., caspase-3). Ca^2+^ plays a vital role in long-term brain damage by increasing free radicals and expression of pro-inflammatory cytokines during brain hypoperfusion (Warner et al., 2004; Daulatzai, 2017). An excessive rise in cytoplasmic Ca^2+^ levels activates the nitric oxide synthase–nitric oxide pathway that fortifies the glutamatergic activation of NMDARs, thereby establishing a vicious cycle of neurodegenerative pathways. Together, free radicals, inflammatory molecules, proteolytic enzymes (e.g., matrix metalloproteinases, myeloperoxidase), and adhesion molecules damage the vascular architecture in the brain, including the blood–brain barrier (BBB) (Wang et al., 2020). These events lead to infiltration of neutrophils and monocytes and activation of macrophages and microglia that further amplify brain damage (Liu and Zhang, 2012). Inhibition of glutamatergic excitatory drive and reestablishment of CBF can alleviate the oxidative and inflammatory insult and protect neurobehavioral functions (Farkas et al., 2002).

Cucurbitacins are steroidal tetracyclic terpenes abundantly found in Cucurbitaceae (e.g., cucumbers, pumpkins, gourds) and several other plant families, such as Scrophulariaceae, Begoniaceae, Primulaceae, Liliaceae, Tropaeolaceae, and Rosaceae (Kaushik et al., 2015). *Cucumis melo* L. and cucurbitacin are Chinese traditional medicines used as antimalarial, emetics, narcotics, and against jaundice (Dhiman et al., 2012; Zhong et al., 2020). Cucurbitacin B and E are the most common and widely studied (Chung et al., 2015). In earlier studies, cucurbitacin showed cytotoxic, antitumor, hepatoprotective, anti-inflammatory, antimicrobial, anthelmintic, cardiovascular, and antidiabetic effects (Dhiman et al., 2012; Chung et al., 2015; Kaushik et al., 2015; Garg et al., 2018; Zhong et al., 2020). Many of the pharmacological activities (e.g., antiproliferative, antiobesity, immunomodulatory, and neuroprotective) of Cucurbitacin E (CuE) are attributed to antioxidant and anti-inflammatory properties (Murtaza et al., 2017; Song et al., 2018; Xie et al., 2020). CuE can modulate several molecular mechanisms (e.g., Janus kinase, signal transducer and activator of transcription, cyclo-oxygenase-2, autophagy, cofilin) that may be exploited for potential benefits in various cerebral disorders (Murtaza et al., 2017; Song et al., 2018). Yuan et al. (2019) showed that pretreatment with cucurbitacin-rich extract of *Cucumis melo* L. inhibited phenylephrine-mediated vasoconstriction, enhanced acetylcholine-mediated vasodilation, and suppressed the angiotensin II-induced increase in systolic blood pressure in mice. Furthermore, anti-inflammatory and antioxidant effects of CuE were observed in carrageenan-induced paw edema and liver damage animal models (Peters et al., 1997; Hussein et al., 2017). Cell culture studies revealed the neuroprotective effects of CuE against 1-methyl-4-phenylpyridinium (MPP^+^) induced Parkinson’s disease by modulating lysosomal-autophagic mechanisms (Arel-Dubeau et al., 2014). These findings indicate that CuE may have therapeutic value against CH states and improve the behavioral outcomes by improving the blood flow and attenuating oxidative and inflammatory cascades in the brain. The present study investigated the neuroprotective and memory-enhancing effects of CuE in permanent 2-vessel occlusion (2-VO)-induced CH in rats.

## Materials and Methods

### Experimental Subjects

The research protocol was accepted by the Animal Ethics Committee of Henan Hospital of Traditional Chinese Medicine (The Second Affiliated Hospital of Henan University of Chinese Medicine) (Henan, China) *vide* approval reference no. ky20210429001 on April 29, 2021. Wistar rats (7–8 month adults) of male sex (body weight range 220 ± 10 g) were housed under a standard laboratory environment. Each rat was housed individually in polyacrylic cages and nurtured using a regular diet and water with unlimited access. Rats were fasted for 12 h before surgery, but water was provided *ad libitum*. The caregivers were blinded to the treatment groups. The experiments on animals were conducted after 2 weeks of an acclimatization phase. Experiments were performed between 0900 and 1600 h.

### Drugs and Chemicals

CuE (mol. weight 556.69, purity >95%) was acquired from Merck (China). Monosodium phosphate (NaH_2_PO_4_), dipotassium phosphate (K_2_HPO_4_), sodium cyanide (NaCN), *p*-Nitrobluetetrazolium chloride (NBT), ethylenediaminetetraacetic acid (EDTA), riboflavin, hydrogen peroxide (H_2_O_2_), Folin–Ciocalteu’s reagent, formaldehyde, bovine serum albumin (BSA), trichloroacetic acid, *n*-butanol, thiobarbituric acid (TBA), acetic acid, 5,5′-Dithiobis-(2-nitrobenzoic acid) (DTNB), sulfosalicylic acid, dimethyl sulfoxide (DMSO), pyridine, and sodium dodecyl sulphate (SDS) (TCI, Shanghai, China); acetylthiocholine (AcTh) iodide, methanol, acetonitrile (HPLC grade), copper sulfate (CuSO_4_), 2,4 dinitrophenylhydrazine (DNPH), sodium-potassium tartrate, sodium nitrite (NaNO_2_), sodium carbonate (Na_2_CO_3_), sulphanilamide, *N*-1-Napthyl ethylenediamine dihydrochloride, 0.5% hexadecyltrimethylammonium bromide (HETAB), 3,3′,5,5′-tetramethylbenzidine (TMB), N, N′-dimethylformamide, heptanes (Fischer-Scientific); catalase (Cayman Chemicals, Ann Arbor, MI, United States) were used. The standards including amino acids *viz.* glutamate and GABA used were procured from Sigma Chemical Co., St. Louis, MO, United States.

### Surgery-CH

The procedure and techniques used in bilateral common carotid arteries occlusion (BCCAo)-induced CH are adopted from previous protocols (Yanpallewar et al., 2005; Bhuvanendran et al., 2019). Atropine sulfate (0.5 mg/kg, *i.p.*) was injected as a preanesthetic treatment. An anesthesia cocktail of ketamine (90 mg/kg) and xylazine (10 mg/kg) was administered through the intraperitoneal (*i.p.*) route in rats. Eye reflex, toe or tail pinch responses were checked to determine the level of general anesthesia. The surgical zone was disinfected using 70% ethanol. A skin incision in the ventral side of the neck (midline) was performed between the sternocleidomastoid and sternohyoid muscles in line with the windpipe (area in the middle of the neck and sternum to reveal the windpipe). Common carotid arteries (both right and left) next to the sternocleidomastoid muscle were isolated vigilantly from the vagosympathetic nerve and adventitial sheath. Bilateral common carotid arteries were permanently double-ligated (*p*-BCCAo) using a sterilized 3–0 silk suture in the CH model. The skin incision was applied with penicillin and 0.5% bupivacaine and then sutured. The first carotid to be ligated, either right or left, was interchanged throughout the experiment (Soria et al., 2013). The temperature of the animals was monitored at regular intervals by a rectal thermometer probe. Body temperature at 37°C ± 0.5°C was maintained during the entire phase using a feedback-adjusted heat-cushion. After sutures, a warm atmosphere (37°C ± 0.5°C) was preserved to avoid hypothermia in animals. Each rat was kept independently in a distinct cage and allowed unhindered access to a semisolid standard diet, and purified water was given. Rats showing reluctance to drinking water postsurgery were given buprenorphine (0.05%, *i.p*) once. Sham rats were exposed to matching surgery without carotid artery ligation.

### Experimental Design

CuE was homogenously suspended in a vehicle (0.5% carboxymethylcellulose; CMC) and given in doses 0.25 and 0.5 mg/kg in rats through the oral route (Lu et al., 2017; Murtaza et al., 2017). The rats were dispersed in six groups in a single blind manner by a random distribution technique (*n* = 12): 1) Sham (S), 2) S + CuE(0.5), 3) CH, 4) CH + CuE(0.25), 5) CH + CuE(0.5), 6) CH + Bay-K8644 + CuE(0.5). Rats were subjected to sham or CH surgery on day 1. CuE was administered for 28 consecutive days after CH or sham procedure on day 1. Bay-K8644 (0.5 mg/kg, *i.p.*, 28 days) (Jackson and Damaj, 2009) was given to rats that were subjected to CuE (0.5 mg/kg) treatment for 28 days and CH on day 1. Animals in sham or CH groups were given the vehicle (0.5% CMC dose volume 5 ml/kg) from days 1–28. The neurological functions and sensorimotor performance were assessed on days 1, 7, 14, 21, and 28. Working memory of rats was evaluated on day 25 using the novel object recognition test (NORT). On days 26 and 27, the animals were exposed to a passive avoidance test. Afterward, whole brain samples were collected for histopathological examination and assessment of biochemical parameters of oxidative stress, such as 8-hydroxy-2′-deoxyguanosine (8-OHdG), thiobarbituric acid reactive substances (TBARS), superoxide dismutase (SOD), catalase, and glutathione (GSH), protein carbonyls, cellular demise *viz.* lactate dehydrogenase (LDH) and caspase-3, acetylcholinesterase (AChE), *γ*-aminobutyric acid (GABA), glutamate, inducible nitric oxide synthase (iNOS), inflammation such as tumor necrosis factor-*α* (TNF-*α*), nuclear factor-kappaB (NF-*κ*B), myeloperoxidase (MPO), and vascular injury such as matrix metalloproteinase 9 (MMP-9).

### Evaluation of Neurological Deficits

Abnormal gait, reflexes, and hemiplegia functions were determined by adopting the standard technique of assessing neurological deficits using a modified 12-point neurological scale (mNSS) (Table 1).

**TABLE 1:** Scoring scale (12-point) for modified neurological severity score test (mNSS) in rats. Each animal was exposed to four different tests (twisting of thorax, forelimb flexion, beam-walk test, and hanging-wire test) and scores added to obtain neurological deficit score (NDS) of an individual animal.

S. No.	Test	Score	Interpretation
1	*Twisting of thorax*	0	No twisting (animal tries to grasp floor)
		1	Wobble to contralateral side (damaged striatum or cortex)
	Each rat was dangled freely by tail (5 s), 25 cm above the floor. Bending of thorax (upward movement of rat toward its tail) was noted	2	Bending upwardly, not touch tail
		3	Bending upwardly, touch tail
2	*Forelimb flexion*	0	Extension of both forepaws towards the ground
		1	Contralateral side flexion of contralateral forepaw with slight flexion
	Rat was suspended by tail and forelimb observed as it reaches floor	2	45° flexion
		3	Distinct 90° flexion
3	*Walk-beam test*	0	Grip normal and no slipping
		1	Grip not firm with irregular slipping
	This method evaluates fore- and hind-limbs motor coordination (limb paralysis). Each rat was placed on the narrow beam (70 cm length × 1.5 cm width) situated at 50 cm height and observed for 15 s	2	Grip completely lost, feet resting on beam
		3	Grip completely lost, feet sagging over beam, immovable
4	*Prehensile traction test*	0	>4 s
		1	≤4 s
	Forelimbs of rat were placed on a nylon rope (70 cm height) with 5 cm thick underneath foam sheet (cutoff period 5 s) and duration of adherence to rope noted	2	≤3 s
		3	≤1 s

### Sensorimotor Test

The rotarod test was used for determining the balance and coordination aspects of sensorimotor performance in rodents. The rats were trained until they were able to run for more than 60 s on a rod rotating at nine rotations per min (rpm). After the training, each rat was placed on the rod and the rotation speed was enhanced incrementally every 10 s from 6 rpm (initial speed) to 30 rpm (final speed) over the course of 50 s. The fall-off latency (s) over the rotating rod before induction of and after CH was noted.

### Evaluation of Working Memory

The assessment of recognition type working memory used discrimination between a familiar and a new article *via* innate probing behavior of rodents in novel object recognition task (NORT) (Ennaceur and Delacour, 1988). An open arena with plywood walls (80 × 40 × 60 cm^3^) and solid-wood objects (three similar copies) of three varied shapes (compact cube, cylinder, and pyramid) having a height of 10 cm were used. Experiments were performed in a noise-attenuated place with uniform lighting conditions. Rats were familiarized prior to testing for 3 successive days by permitting search of the vacant arena for 5 min duration. In the training trial, two alike objects were situated at indiscriminately designated divergent angles of the arena with a 10 cm gap from the plywood walls. Each animal was positioned in the center of the arena facing at a 90° angle away from the alike objects. Afterward, time expended by the animal to scrutinize the individual objects (z1 and z2) was observed (cutoff period 5 min). In probing behavior, sniffing, touching, or investigating the object within a 2 cm zone was acceptable. After 5 min of intertrial pause, the retention trial was initiated. A new article replaced one of the former articles, and time expended in investigation of each article (z3 and y) by the animal was observed. To decline plausible partiality caused by fondness for specific places or articles, complete amalgamations and settings within the arena were compensated. After every single trial, olfactive signs were circumvented by wiping the apparatus using 20% ethanol. The discrimination index (DI) mirrors the variation in time expended examining the familiar article and the new article (y—z3) that represents the memory. DI was quantified using formula (y—z3)/entire investigation time during the retention trial.

### Passive Avoidance (PA) Test

Restriction over the impulsive investigative habit of rodents is used in this inhibitory avoidance test in which the animal adapts to circumvent the aversive stimulus given in the form of a foot shock (1.0 mA for 5 s). The step-through PA instrument is made of twin alike dimensions light–dark compartments (23 × 22 × 23 cm^3^) separated by a guillotine gate. The dark box consists of plywood walls, and the light box consists of plexiglass walls having a light source (bulb 60 W). A grid floor arrangement *viz.* 3.1 mm stainless-steel rods placed at a distance 8 mm away from each other made the floor of the dark compartment to issue a scrambled electric shock to rodents. A noise-attenuated place was used to perform the trials. During the training trial (day 26), an individual rat was positioned in the light compartment with the snout contrary to the guillotine gate, which was opened after 10 s, and the rat was permitted to pass into the dark compartment. The time taken to pass into the dark compartment from the light compartment was determined using a digital stopwatch. Afterward, the guillotine gate was shut, and an inevitable foot shock ensued. The animal escaped from the dark compartment after 15 s of termination of shock and placed in its home cage. A retrieval test was performed 24 h post training trial (i.e., on day 27) with an identical practice excepting the foot shock. The step-through latency time (STL) to pass into the dark compartment was noted during acquisition and retrieval trials for the individual rat, giving a 300 s cutoff period (Gimenez De Bejar et al., 2017).

### Estimation of Biochemical Parameters

The rats were euthanized by the cervical dislocation method (Sodium pentobarbitone 150 mg/kg). Instantly, the complete brain was harvested and bathed with freezing sterile isotonic normal saline. The brain was homogenized using tissue homogenizer in ice-cold 50 mM sodium-phosphate phosphate buffer (pH 7.40) to prepare 10% w/v brain homogenate. Subsequently, a clear supernatant was obtained by centrifuging the whole brain homogenate for 15 min at 4°C at 12,000 × *g* force. The clear supernatant was separated for evaluation of biochemical parameters.

#### Lipid Peroxidation

For assessment of thiobarbituric acid reactive substances (TBARS) (Ohkawa et al., 1979) the assay blend (final volume 4 ml) containing 0.10 ml homogenized brain, 1.50 ml TBA (0.8%), 1.50 ml glacial acetic acid (20%, pH 3.50), 0.20 ml SDS (8.10%), and 0.70 ml purified water was boiled at 95°C for 1 h, *n*-butanol/pyridine (5 ml in 15:1 ratio) was added into test tubes, assay blend centrifuged at 4000 × *g* (10 min), and supernatant was separated. Optical density (O.D.) of the malondialdehyde-thiobarbituric acid (MDA-TBA_2_) chromophore was determined at the wavelength (*λ*_max_ = 532 nm) using a two-beam UV1700 spectrophotometer (Shimadzu, Japan). The molar extinction coefficient (*ε*) = 1.56 × 10^5^ /M/cm at *λ*_max_ = 532 nm was used to estimate TBARS expressed as nmol per mg protein.

#### Glutathione Levels

Glutathione was assessed adopting the technique of Ellman (1959). Briefly, the test blend comprising homogenate supernatant (1.0 ml) and 4% sulfosalicylic acid (1 ml) was centrifuged for 10 min (4°C) at 2000 × *g* force. Subsequently, 2.7 ml sodium-potassium phosphate buffer (50.0 mM, pH 7.80) and 0.20 ml DTNB (0.10 mM, pH 8.0) was mixed with 0.10 ml separated supernatant. Glutathione (*µ*mol GSH per mg protein) was enumerated spectrophotometrically (*λ*_max_ = 412 nm) by using *ε* = 1.36 × 10^4^ /M/cm at *λ*_max_ = 532 nm.

#### Superoxide Dismutase Activity

The activity of superoxide dismutase (SOD) was appraised by following the technique of Winterbourn et al. (1975). Briefly, the test tubes (final volume 3 ml) containing 0.05 ml supernatant of homogenized whole brain, 0.05 ml riboflavin (0.12 mM), 0.10 ml NBT (1.50 mM), 0.20 ml of 0.10 M EDTA (0.30 mM NaCN), and Na^+^/K^+^ PO_4_^3-^ buffer (67.0 mM, pH 7.81) *q.s.* were illuminated for 13 ± 3 min below a 100 W fluorescent-tube (Bajaj^®^) and O.D. variability (*λ*_max_ = 560 nm) noted. The SOD in the sample impedes the reduction of NBT by O_2_− and formazan synthesis. SOD activity (*µ*mol NBT reduced per min per mg protein) is quantified using *ε* (formazan) = 15,000 /M/cm.

#### Catalase Activity

For estimation of catalase activity, the disparity in O.D. of the analyte (3.0 ml) containing 0.05 ml homogenized brain supernatant, 1.10 ml H_2_O_2_ (0.02 M) in Na^+^/K^+^ PO_4_^3−^ buffer (pH 7.80, 0.05 M), and 1.85 ml of 0.05 M Na^+^/K^+^ phosphate buffer (pH 7.0) was noted at *λ*_max_ = 240 nm (Claiborne, 1985). Catalase activity (*µ*mol H_2_O_2_ decomposed per min per mg protein of brain) was enumerated by means of *ε* = 43.6 /M/cm.

#### Acetylcholinesterase Activity

Acetylcholinesterase (AChE) activity was determined by following the technique of Ellman et al. (1961). Briefly, the test tube volume was made of 0.10 ml acetylthiocholine (AcTh) iodide (1.585 M), 0.10 ml DTNB (0.01 M), 3 ml Na^+^/K^+^ PO_4_^3−^ buffer (0.10 M, pH 8.0), and 0.05 ml supernatant. Variation in O.D. was observed at *λ*_max_ = 412 nm by employing twin-beam UV-spectrophotometer. The activity of AChE (*µ*mol acetylthiocholine iodide hydrolyzed per min per mg protein) was determined utilizing *ε* = 1.36 × 10^4^ /M/cm at *λ*_max_ = 412 nm.

#### Lactate Dehydrogenase Activity

The lactate dehydrogenase activity (*µ*mol NADH oxidized/min/mg protein) was examined using a technique given by Horecker and Kornberg (1948) using *ε* = 6220 M^−1^cm^−1^ at *λ*_max_ = 340 nm. The total reaction mixture (3 ml) contained 1 ml of 0.2 M Tris-HCl buffer (pH 7.4), 0.15 ml of 0.1 M KCl, 0.15 ml of 50 mM sodium pyruvate, 0.20 ml of 2.4 mM NADH and supernatant. A decrease in extinction for 2 min at 25°C was measured, and the result was expressed in micromole NADH oxidized/min/mg protein.

#### Total Protein

The total protein content (mg per mL of homogenate) was quantified using a typical curve of BSA with a concentration range 0.2–2.4 mg/ml. The test blend was arranged using 0.25 ml homogenate supernatant, Lowry’s reagent (5.0 ml), Na^+^/K^+^ PO_4_^3-^ buffer (1.0 ml), and Folin–Ciocalteu reagent (0.50 ml of 1.0 N). O.D. was determined at *λ*_max_ = 650 nm (Lowry et al., 1951).

### Estimation of Myeloperoxidase Activity

A pellet was used for determination of myeloperoxidase (MPO) activity (units per mg protein) used as a biomarker of inflammatory neutrophil extravasation (Grisham et al., 1990). The pellet is homogenized in 10 volumes of 50 mM potassium phosphate buffer (ice cold, pH 6.2) added with 0.5% hexadecyltrimethylammonium bromide (HETAB) and 10 mM EDTA. MPO catalyzes the oxidation of 3,3′,5,5′-tetramethylbenzidine (TMB) by hydrogen peroxide to produce a blue chromophore that has *λ*_max_ = 655 nm. The above homogenate is mixed with 0.5 ml reaction mixture having 80 mM potassium phosphate buffer (pH 5.4), HETAB (0.5% w/v), and TMB (1.6 mM) added as a stock solution (10 mM) prepared in N, N′-dimethylformamide. The reaction is then heated to 37°C and started with hydrogen peroxide (0.3 mM). Each test tube is incubated for 3 min (37°C). The reaction is ended by sequential addition of catalase (20 *μ*g/ml) and 0.2 M sodium acetate (2 ml, pH 3.0) at 4-min intervals and ice cooled. Centrifugation can be used to eliminate extraneous membranous material that can affect spectrophotometric analysis. The O.D. of each reaction test tube is noted at *λ*_max_ = 655 nm and subsequently corrected by subtracting the blank value. One unit of activity is the quantity of enzyme existing that produces an alteration in O.D. per minute of 1.0 at 37°C in concluding reaction volume comprising sodium acetate. MPO activity was calculated: MPO activity (U/mg protein) =  N/tissue weight with N = 10 × (change in O.D./min)/volume of supernatant taken in final reaction.

### Estimation of Protein Carbonyl Content

Brain homogenates were diluted to 750–800 μg/ml of protein in individual samples, and 1 ml of diluted sample was added with 10.1 mM DNPH (0.2 ml) or the same volume of 2 M HCl. The sample was incubated at 25°C for 1 h in a dark environment. Subsequently, 0.6 ml of denaturing buffer (150 mM sodium phosphate buffer, pH 6.8 with 3% SDS), 1.8 ml of heptanes (99.5%), and 1.8 ml of ethanol (99.8%) were added. The sample was vortexed for 40 s and centrifuged at 4000 × *g* force for 15 min. Protein was secluded from the interface and washed twice with 1 ml of ethyl acetate/ethanol 1:1 (v/v). Separated protein was suspended in 1 ml of denaturing buffer and absorbance noted at *λ*_max_ = 370 nm spectrophotometrically. Protein carbonylation was quantified using *ε* = 22,000 /M/cm (Reznick and Packer, 1994; Yan et al., 1995).

### HPLC-FLD Analysis of Neurotransmitters

The brain was swiftly harvested on frozen ice, weighed, and homogenized using 15 volumes of methanol/water (85:15 v/v). The homogenate was centrifuged (7800 × *g* for 15 min at 4°C) and supernatants detached, filtered by means of a cellulose membrane of pore size 0.22 µm, and then kept at −20°C until derivatization for GABA/glutamate analysis. The filtered supernatant (10 μL) was diluted with deionized water (990 µL). The OPA derivatization technique was used for assessment of GABA and glutamate. Standard (glutamate or GABA) or supernatant (100 µL) was subjected to precolumn derivatization in microcentrifuge tubes (Eppendorf) using OPA solution (22 µL) at room temperature in the dark for 10 min. The OPA solution consisted of methanolic OPA (5 mg/ml), 75 µL borate buffer (pH 9.9), and 5 µL 3-mercaptopropionic acid. The derivatization product (20 µL) was inoculated into the column (C18 column; 5 μm, 4.6 × 250 mm) of HPLC system (Waters) with fluorescence detection (Agilent 1260 Infinity FLD G1321C) coupled with an LC-10 AD pump. The mobile phase used comprised 0.05 M sodium acetate, tetrahydrofuran, and methanol (HPLC grade) (49:1:50 v/v/v) (pH 4.1) and filtered through 0.22 µm and vacuum degassed before disseminated in HPLC at flow rate 0.05–0.1 ml/min. The column temperature was upheld at 23°C–27°C. Compounds were eluted isocratically over a 15-min runtime at a flow rate of 1 ml/min. The fluorescent detector was set at an excitation wavelength of *λ*_max_ = 337 nm and an emission wavelength of *λ*_max_ = 454 nm. The concentrations of neurotransmitters were measured by means of external standards and the zone under the peak procedure. The peak zones were determined by injecting serial dilutions of standards. Peak zones (upright axis) versus matching concentrations (flat axis) of individual discrete amino acid were plotted to obtain a linear standard arc and used to quantify the concentrations in samples. Concentration of glutamate and GABA reported as *µ*g/mg of the brain tissue.

### Enzyme-Linked Immunosorbent Assay

A double antibody sandwich ELISA method was employed to measure the TNF-*α* (#KB3145, Krishgen Biosystems), NF-*κ*B (#K11-0,288, KinesisDX, United States), MMP-9 (E-EL-R3021, Elabscience), caspase-3 (#E4592, Biovision), 8-hydroxy-2′-deoxyguanosine (#ADI-EKS-350, Enzo LifeSciences), and iNOS (#E4649, Biovision) levels in the rat brain samples. The assay procedure specified in the instruction brochure provided in the kits was appropriately followed. Briefly, whole brain tissue was homogenized and then centrifuged at 2500 × *g* for 20 min. Supernatant was taken and filled in wells (12 × 8 wells) that were precoated with rat monoclonal antibodies. The plates were incubated at 37°C for 1 h. Biotin-labeled detection antibody followed by streptavidin-horseradish peroxidase were added and plates were covered and again incubated. A bluish coloration was obtained by the addition of chromogenic solution A/B or TMB substrate. The reaction was stopped by adding stop solution, and instantly (within 15 min) O.D. was observed at a wavelength *λ*_max_ = 450 nm by using an ELISA reader (iMARK, BIORAD). A standard curve of different biomarkers (concentrations standard rat TNF-*α* 450, 225, 56.25, 28.13, 14.06, 7.03, and 3.51 pg/ml; NF-*κ*B 12, 6, 3, 1.5, and 0.75 ng/ml; MMP-9 7.81, 15.63, 31.25, 62.5, 125, 250, and 500 ng/ml; caspase-3 and iNOS 0.313, 0.625, 1.25, 2.5, 5, 10, and 20 ng/ml, 8-OHdG 0.94, 1.875, 3.75, 7.5, 15, 30, and 60 ng/ml) was plotted to estimate TNF-*α* (pg/ml), NF-*κ*B (ng/ml), MMP-9 (ng/ml), caspase-3 (ng/ml), iNOS (ng/ml), and 8-OHdG (ng/ml) in the samples.

### Brain Histopathology

Animals were deeply anesthetized and intracardially (*via* left ventricle) perfused with 10% neutral buffered formalin solution (10% NBF) by using a gravity fed perfusion setup. The cortex were fixed for 6 days (4°C) in 10% neutral buffered formalin with 0.05% sodium azide (pH 7.0) in a fixative-to-tissue ratio of 10:1. A 70% ethanol was used as storage medium (4°C) for the previously fixed brain tissues. A rotary microtome was used to section out thin slices (5.0 μm) that were stained using hematoxylin and eosin (H&E) dye. Permanent slides were prepared using DPX-resin mounting medium and subsequently coverslipped. These slides were scrutinized using light microscopy at ×40 magnifications. In histomorphometric analysis, cortical neuron densities (per µm^2^) were determined by counting viable neurons using ImageJ software (NIH Image 1.61; National Institute of Health; Bethesda, MD).

### Statistical Analysis

Data were analyzed by a skilled experimenter blinded to miscellaneous treatments received by diverse clusters of rats. Data from the PA test, NORT, biochemical and histomorphometry parameters were analyzed using one-way ANOVA and Tukey’s honestly significant difference (HSD) *post hoc* test. Grubb’s test was applied to eradicate likely outliers even though no outliers were detected. To determine the typical distribution of variables, the Kolmogorov–Smirnov test was used. Levene’s test was applied to test the homogeneity of variance (HOV). One-way ANOVA was applied to compare the means of normally distributed variables. If the variance was homogeneous (*p* > .05, Levene’s test) and the outcomes of one-way ANOVA were substantial (*p* < .05, F-statistic), Tukey’s HSD *post hoc* test was used for multiple comparisons. If the variance was unequal (*p* < .05, Levene’s test), Welch’s ANOVA was used (F′-statistic), and if the matching *p* was <.05, the Games–Howell technique was used for the *post hoc* analyses. The *post hoc* tests were only applied when ANOVA results were significant (*p* < .05). Box and whisker plots (Tukey) demonstrate mean (+), median (bold horizontal line), quartiles (box), and total range (whisker). Data from mNSS and rotarod were analyzed using two-way ANOVA. When two-way ANOVA generated a significant interaction, then Bonferroni’s *post hoc* test was applied. Results of mNSS and rotarod expressed as mean ± standard deviation (S.D.). Statistical significance was considered at *p* < .05.

## Results

### CuE Attenuated CH-Induced Neurological and Sensorimotor Deficits

**FIGURE 1:**
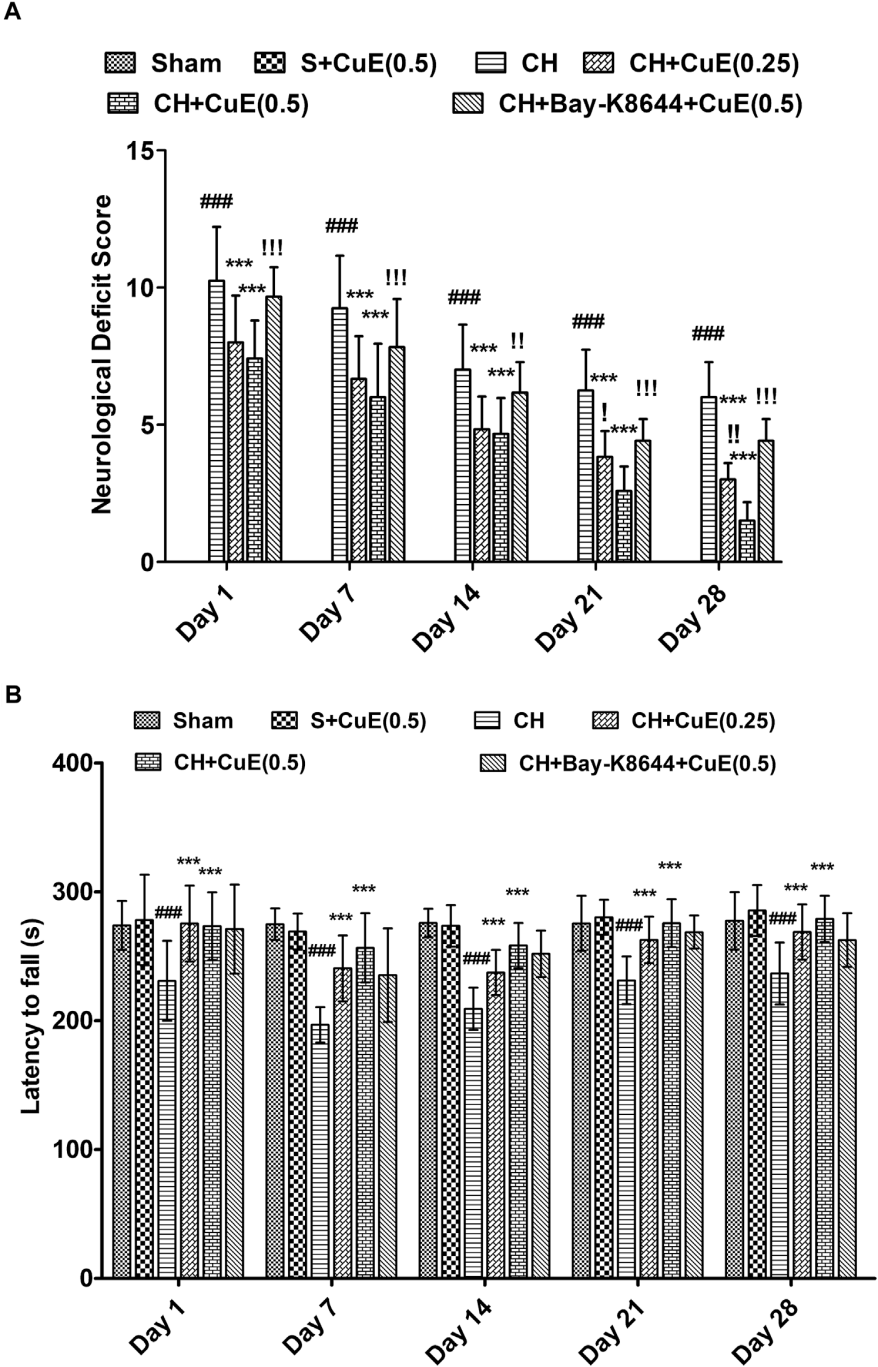
CuE, 0.25 and 0.5 mg/kg, *p.o.*, post-treatment for 28 days attenuated neurological and sensorimotor deficits in rats subjected to CH on day 1. Neurological deficit scores (12-point scale) **(A)** and latency to fall (s) in rotarod test **(B)** evaluated on different days. Bay-K8644 significantly attenuated CuE-induced decrease in mNSS. Data expressed as mean ± S.D. (*n* = 12). ^###^ (*p* < .001) *vs.* Sham (S) group; ^***^ (*p* < .001) *vs.* CH group; (*p* < .05), (*p* < .01), (*p* < .001) *vs.* CH + CuE(0.5) group

Rats exposed to CH on day 1 showed substantial neurological and sensorimotor deficits (days 1, 7, 14, 21, and 28, *p* < .001) relative to sham rats. CuE (0.25 and 0.5 mg/kg) post-treatment in rats for 28 days attenuated CH-induced neurological deficits (*p* < .001) [F_(20,330)_ = 12.63, *p* < .001] (Figure 1A) and also improved sensorimotor performance (*p* < .001) [F_(20,330)_ = 1.41, *p* < .001] (Figure 1B) compared with rats that were given CH and vehicle treatments alone. Interestingly, administration of Bay-K8644 (Ca^2+^ agonist) significantly attenuated CuE (0.5 mg/kg) induced decline in mNSS (day 1 *p* < .001, day 7 *p* < .001, day 14 *p* < .01, day 21 *p* < .001, and day 28 *p* < .001) in rats exposed to CH relative to rats that were subjected to CuE (0.5 mg/kg) and CH surgery. Furthermore, CuE (0.5 mg/kg) showed a dose-dependent decrease in mNSS (day 21 *p* < .05; day 28 *p* < .01) in comparison with CuE (0.25 mg/kg) in rats subjected to CH.

### CuE Attenuated CH-Induced Memory Deficits

**FIGURE 2:**
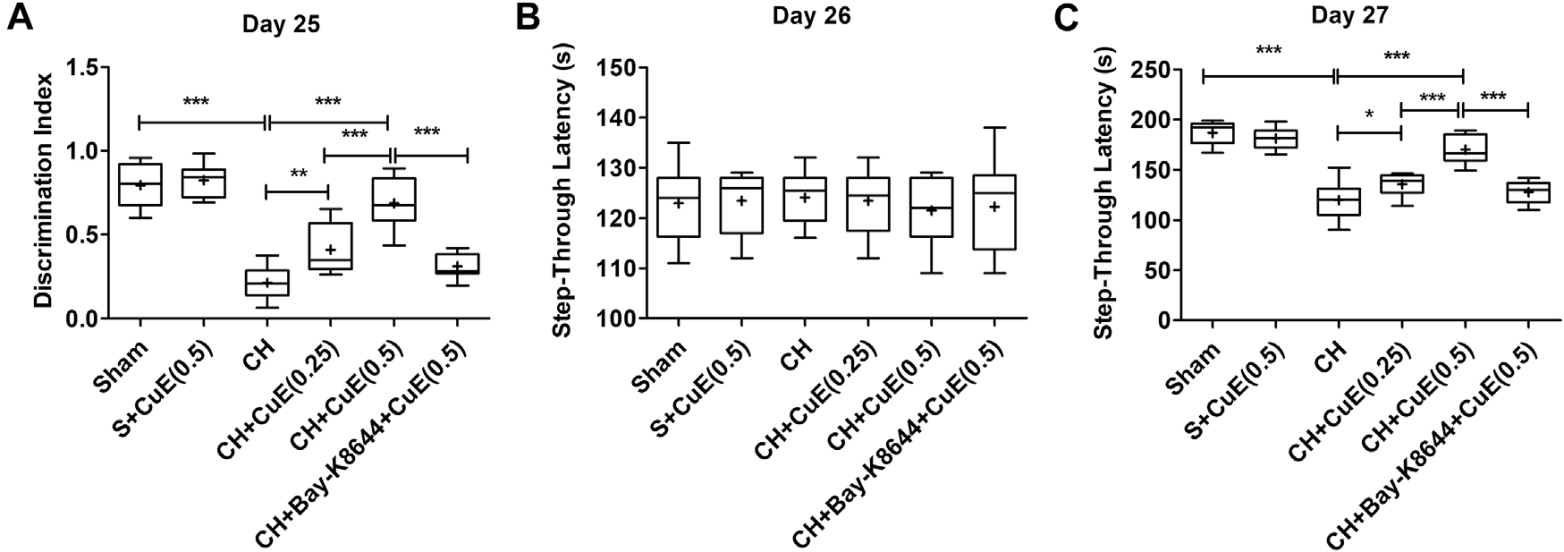
Post-treatment with CuE, 0.25 and 0.5 mg/kg, *p.o.*, decreased CH-induced loss of memory in rats. In a novel object recognition task, animals treated with CuE exhibited increase in discrimination index **(A)**. STL (s) during acquisition trials in PA tests showed no significant difference **(B)**, CuE significantly decreased retention trial STL **(C)**. Bay-K8644 significantly attenuated increase in DI and STL by CuE. Box and whisker plots (Tukey) show mean (+), median (bold horizontal line), quartiles (box), and total range (whisker) (*n* = 12). ^*^(*p* < .05), ^**^ (*p* < .01), ^***^ (*p* < .001)

Estimation of DI in NORT on day 25 revealed that permanent 2-VO on day 1 debilitated recognition type of working memory in rats. Rats subjected to CH showed a considerable decrease (*p* < .001) in DI relative to sham [F_(5,71)_ = 60.52, *p* < .001]. CuE (0.25 and 0.5 mg/kg) post-treatment in rats subjected to CH attenuated the memory deficits (*p* < .01, *p* < .001) relative to rats that were exposed to CH and vehicle administrations (Figure 2A). In the PA test, STL was evaluated to determine the effects of CuE on memory of rats subjected to CH. In acquisition trials, no significant intergroup variation in day 26 STL of rats was noted in the PA test [F_(5,71)_ = 0.2264, *p* > .05] (Figure 2B). In the retrieval trials (day 27), a significant increase (*p* < .001) in the STL (Figure 2C) was noted in rats subjected to CH in reference to vehicle-treated sham. CuE (0.25 and 0.5 mg/kg) treatment attenuated the CH-triggered decline in the STL (*p* < .05, *p* < .001) with respect to the rats that were subjected to CH and vehicle treatments only [F_(5,71)_ = 62.67, *p* < .001]. CuE (0.5 mg/kg) significantly enhanced the DI (*p* < .001) and STL (*p* < .001) in comparison with CuE (0.25 mg/kg) in *p*-BCCAo operated rats. Bay-K8644 (Ca^2+^ agonist) significantly attenuated (*p* < .001) CuE (0.5 mg/kg) induced improvement in DI and STL relative to CuE (0.5 mg/kg) in *p*-BCCAo operated rats.

### CuE Decreased Brain Oxido-Nitrosative Stress Against CH

**FIGURE 3:**
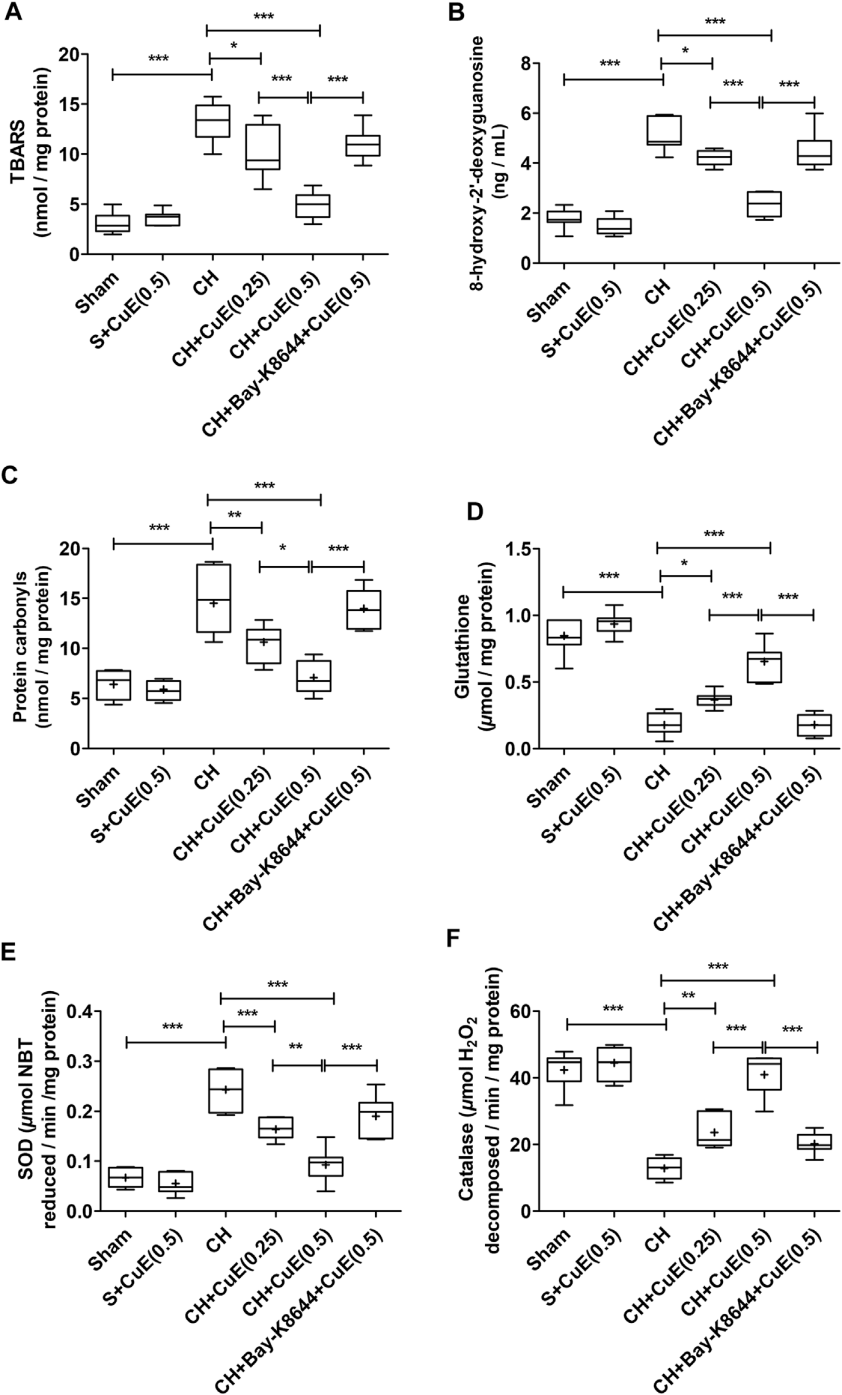
CuE post-treatment (0.25 and 0.5 mg/kg, *p.o.*) for 28 days decreased oxido-nitrosative stress against CH. CuE significantly decreased the brain **(A)** lipid peroxidation (TBARS) level, **(B)** 8-hydroxy-2' -deoxyguanosine (8-OHdG) content, and **(C)** protein carbonyls levels. A significant increase in **(D)** GSH content, **(E)** SOD activity, and **(F)** catalase activity was observed in CuE-treated rats subjected to CH on day 1. Bay-K8644 attenuated antioxidant activity of CuE. Box and whisker plots (Tukey) show mean (+), median (bold horizontal line), quartiles (box), and total range (whisker) (*n* = 7). ^*^ (*p* < .05), ^**^ (*p* < .01), ^***^ (*p* < .001)

Rats subjected to vehicle treatments and CH displayed a substantial (*p* < .001) upsurge in the brain lipid peroxidation (TBARS content), 8-OHdG, and protein carbonyls, and decrease of endogenous antioxidants (GSH, SOD, and catalase activities) relative to vehicle-treated sham rats (Figure 3). CuE (0.25 and 0.5 mg/kg) post-treatment for 28 days daily in rats exposed to CH attenuated the lipid peroxidation (*p* < .05, *p* < .001) [F_(5,41)_ = 45.64, *p* < .001], 8-OHdG (*p* < .05, *p* < .001) [F_(5,41)_ = 59.65, *p* < .001], and protein carbonyls (*p* < .01, *p* < .001) [F_(5,41)_ = 26.94, *p* < .001], and significantly enhanced the GSH (*p* < .05, *p* < .001) [F_(5,41)_ = 79.92, *p* < .001], SOD (*p* < .001, *p* < .001) [F_(5,41)_ = 41.61, *p* < .001], and catalase (*p* < .01, *p* < .001) [F_(5,41)_ = 58.36, *p* < .001] activities in relation to rats that had undergone CH and vehicle treatments. CuE (0.5 mg/kg) post-treatment for 28 days caused a dose-dependent decline in TBARS (*p* < .001), 8-OHdG (*p* < .001), protein carbonyls (*p* < .05), and increase in GSH (*p* < .001), SOD (*p* < .01), and catalase (*p* < .001) in the brain with respect to CuE (0.25 mg/kg) post-treatment for the same duration in rats operated to *p*-BCCAo on day 1. Bay-K8644 (Ca^2+^ agonist) significantly attenuated (*p* < .001) CuE (0.5 mg/kg) induced decline in TBARS, 8-OHdG, protein carbonyls, and increase in antioxidants (GSH, SOD, catalase) relative to CuE (0.5 mg/kg) in *p*-BCCAo operated rats.

### CuE Attenuated CH-Triggered Inflammation in the Brain

**FIGURE 4:**
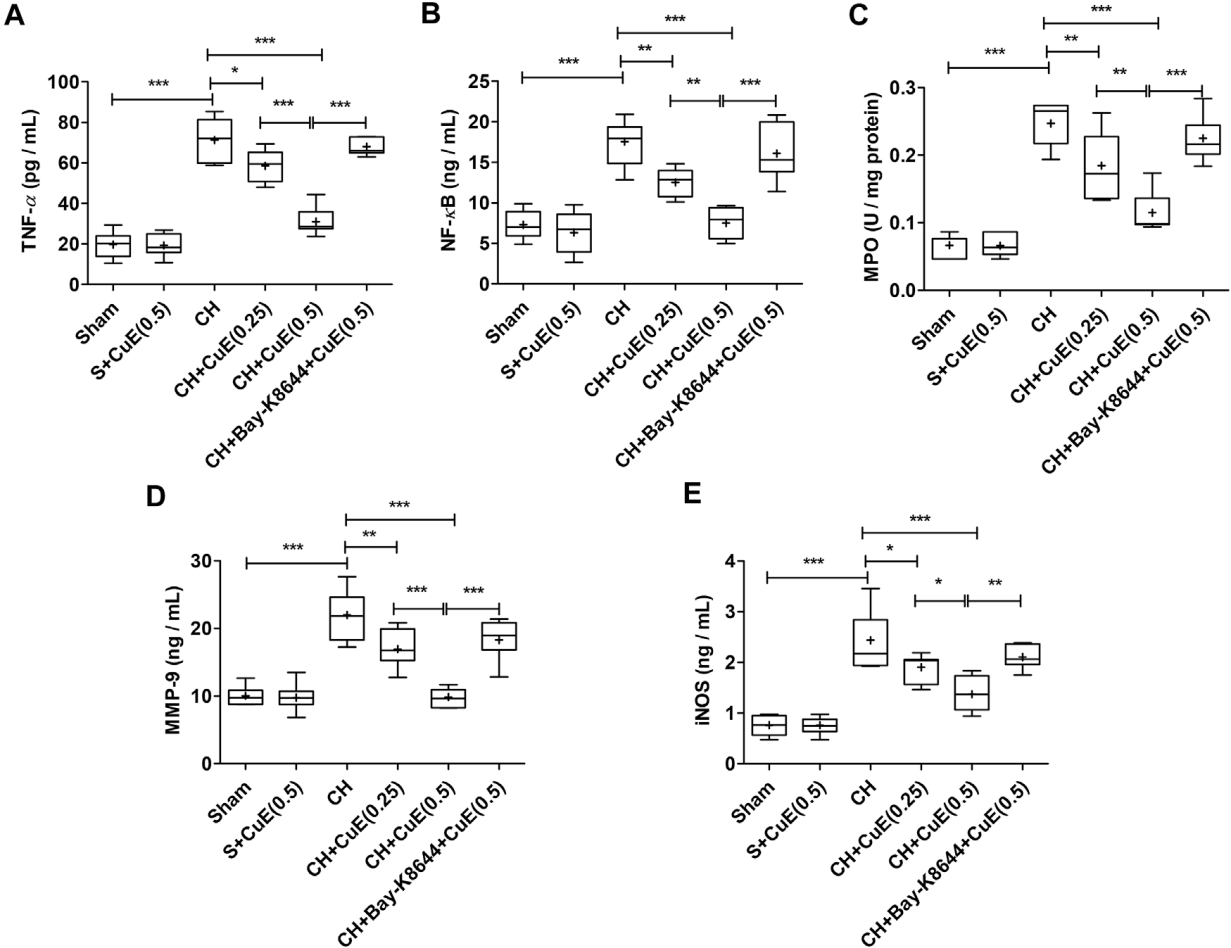
CuE post-treatment (0.25 and 0.5 mg/kg, *p.o.*) for 28 days decreased inflammatory cascade against CH. CuE significantly lowered **(A)** TNF-*α*, **(B)** NF-*κ*B level, **(C)** MPO activity, **(D)** MMP-9, and **(E)** iNOS in the brain of rats exposed to CH on day 1. Bay-K8644 attenuated anti-inflammatory activity of CuE. Box and whisker plots (Tukey) show mean (+), median (bold horizontal line), quartiles (box), and total range (whisker) (*n* = 7). ^*^ (*p* < .05), ^**^ (*p* < .01), ^***^ (*p* < .001)

Data from ELISA showed a significant rise (*p* < .001) in inflammatory cytokines (TNF-*α*, NF-*κ*B, MPO, MMP-9, and iNOS) in the brain of rats in response to CH when juxtaposed with sham rats. In the present study, CuE (0.25 and 0.5 mg/kg) post-treatment for 28 successive days attenuated the CH-induced increase in TNF-*α* (*p* < .05, *p* < .001) [F_(5,41)_ = 81.95, *p* < .001], NF-*κ*B (*p* < .01, *p* < .001) [F_(5,41)_ = 27.97, *p* < .001], MPO (*p* < .01, *p* < .001) [F_(5,41)_ = 43.84, *p* < .001], MMP-9 (*p* < .01, *p* < .001) [F_(5,41)_ = 31.52, *p* < .001], and iNOS (*p* < .05, *p* < .001) [F_(5,41)_ = 33.60, *p* < .001] in the brain of rats with respect to rats that were exposed to CH and vehicle treatments (Figure 4). Bay-K8644 (Ca^2+^ agonist) significantly attenuated CuE (0.5 mg/kg) induced decline in TNF-*α* (*p* < .001), NF-*κ*B (*p* < .001), MPO (*p* < .001), MMP-9 (*p* < .001), and iNOS (*p* < .01) relative to CuE (0.5 mg/kg) in *p*-BCCAo operated rats. CuE (0.5 mg/kg) post-treatment caused substantial reduction in TNF-*α* (*p* < .001), NF-*κ*B (*p* < .01), MPO (*p* < .01), MMP-9 (*p* < .001), and iNOS (*p* < .05) relative to CuE (0.25 mg/kg) in *p*-BCCAo operated rats.

### CuE Attenuated CH-Triggered Cell Death in the Brain

**FIGURE 5:**
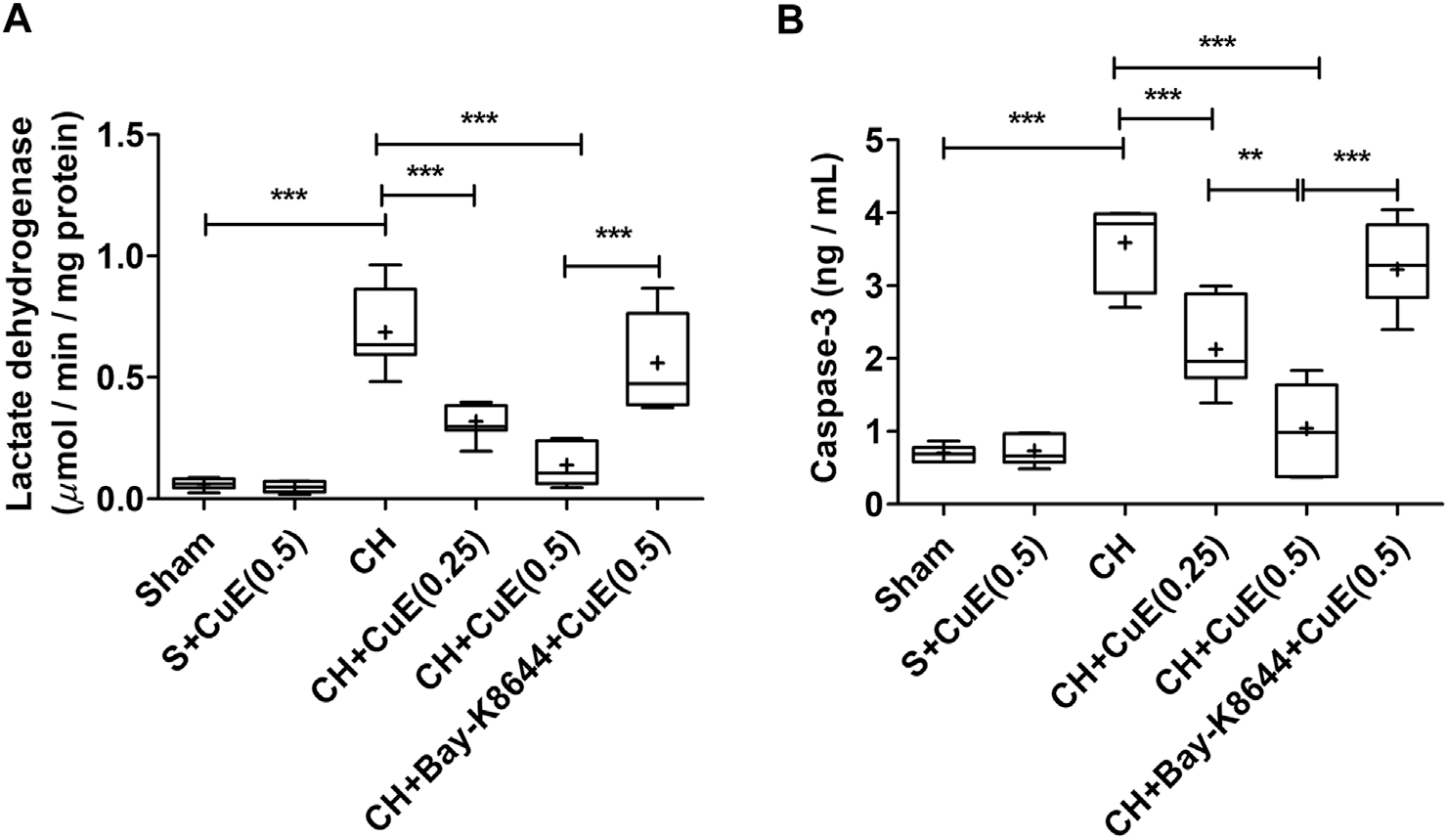
CuE post-treatment (0.25 and 0.5 mg/kg, *p.o.*) for 28 days attenuated cell death against CH. CuE significantly lowered **(A)** LDH activity and **(B)** caspase-3 levels in the brain of rats exposed to CH on day 1. Bay-K8644 attenuated pro-survival effects of CuE. Box and whisker plots (Tukey) show mean (+), median (bold horizontal line), quartiles (box), and total range (whisker) (*n* = 7). ^*^ (*p* < .05), ^**^ (*p* < .01), ^***^ (*p* < .001)

LDH activity and caspase-3 were quantified to assess the extent of cell death in the brain of rats. In the present study, LDH activity and caspase-3 contents were substantially enhanced (*p* < .001) in the brain of rats that were subjected to CH when juxtaposed with sham rats. CuE (0.25 and 0.5 mg/kg) post-treatment for 28 successive days considerably attenuated (*p* < .001, *p* < .001) the LDH activity [F_(5,41)_ = 39.55, *p* < .001] (Figure 5A) and caspase-3 content [F_(5,41)_ = 46.58, *p* < .001] (Figure 5B) in rats subjected to CH with respect to rats that were exposed to CH and vehicle treatments. CuE (0.5 mg/kg) post-treatment caused substantial reduction (*p* < .01) in caspase-3 levels relative to CuE (0.25 mg/kg) in rats exposed to CH. Bay-K8644 (Ca^2+^ agonist) significantly attenuated (*p* < .001) CuE (0.5 mg/kg) induced decline in LDH activity and caspase-3 relative to CuE (0.5 mg/kg) in *p*-BCCAo operated rats.

### CuE Attenuated Acetylcholinesterase Activity and Ameliorated Neurotransmitter Levels in the Brain of Rats Subjected to CH

**FIGURE 6:**
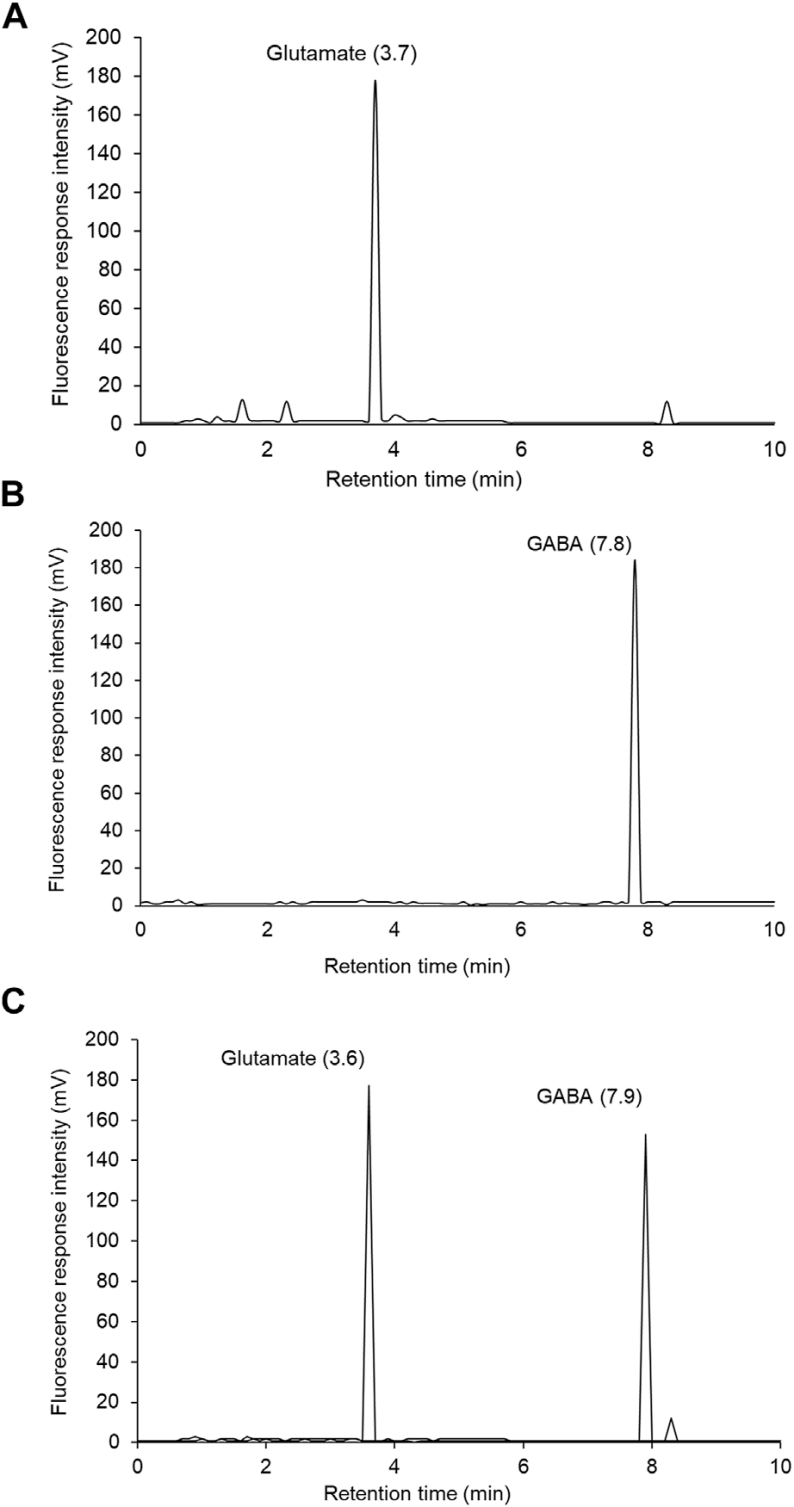
Representative HPLC-FLD chromatograms of glutamate and GABA OPA derivatives: **(A)** glutamate standard solution (0.75 µg), **(B)** GABA standard solution (0.1 µg), **(C)** brain sample showing glutamate and GABA peaks.

**FIGURE 7:**
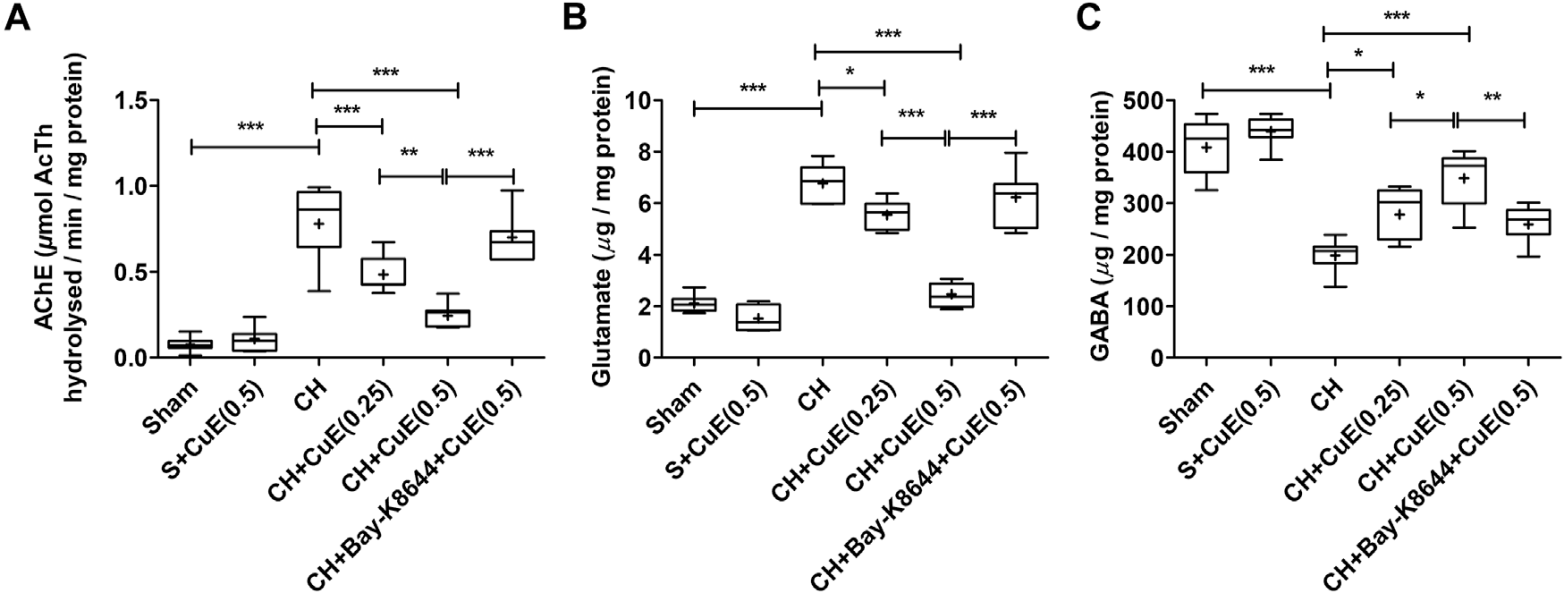
CuE post-treatment (0.25 and 0.5 mg/kg, *p.o.*) for 28 days decreased AChE activity and ameliorated neurotransmitter levels against CH. CuE significantly lowered **(A)** AChE activity, **(B)** glutamate levels, and enhanced **(C)** *γ*-aminobutyric acid (GABA) level in the brain of rats exposed to CH on day 1. Bay-K8644 attenuated amelioration of neurotransmitters by CuE. Box and whisker plots (Tukey) show mean (+), median (bold horizontal line), quartiles (box), and total range (whisker) (*n* = 7). ^*^ (*p* < .05), ^**^ (*p* < .01), ^***^ (*p* < .001)

Biochemical analysis revealed a substantial (*p* < .001) upsurge in brain acetylcholinesterase (AChE) activity in rats exposed to CH and vehicle treatments observed with respect to vehicle-treated sham rats. In HPLC-FLD chromatograms (Figure 6), we detected a significant increase in glutamate levels and decline in GABA levels in rats exposed to CH and vehicle treatments observed with respect to vehicle-treated sham rats. Post-treatment with CuE (0.25 and 0.5 mg/kg) for 28 successive days diminished the AChE activity (*p* < .001, *p* < .001) [F_(5,41)_ = 44.51, *p* < .001] (Figure 7A), glutamate levels (*p* < .05, *p* < .001) [F_(5,41)_ = 90.03, *p* < .001] (Figure 7B), and enhanced the GABA levels (*p* < .05, *p* < .01) [F_(5,41)_ = 32.52, *p* < .001] (Figure 7C) in the brain of rats subjected to CH in comparison to rats that were exposed to CH and vehicle treatments only. CuE (0.5 mg/kg) post-treatment caused substantial reduction in AChE activity (*p* < .01), glutamate level (*p* < .001), and enhanced GABA content (*p* < .05) relative to CuE (0.25 mg/kg) in rats exposed to CH. Bay-K8644 (Ca^2+^ agonist) significantly attenuated CuE (0.5 mg/kg) induced decline in AChE activity (*p* < .001), glutamate level (*p* < .001), and increase in GABA (*p* < .01) relative to CuE (0.5 mg/kg) in *p*-BCCAo operated rats.

### CuE Attenuated CH-Triggered Neurodegenerative Changes

**FIGURE 8:**
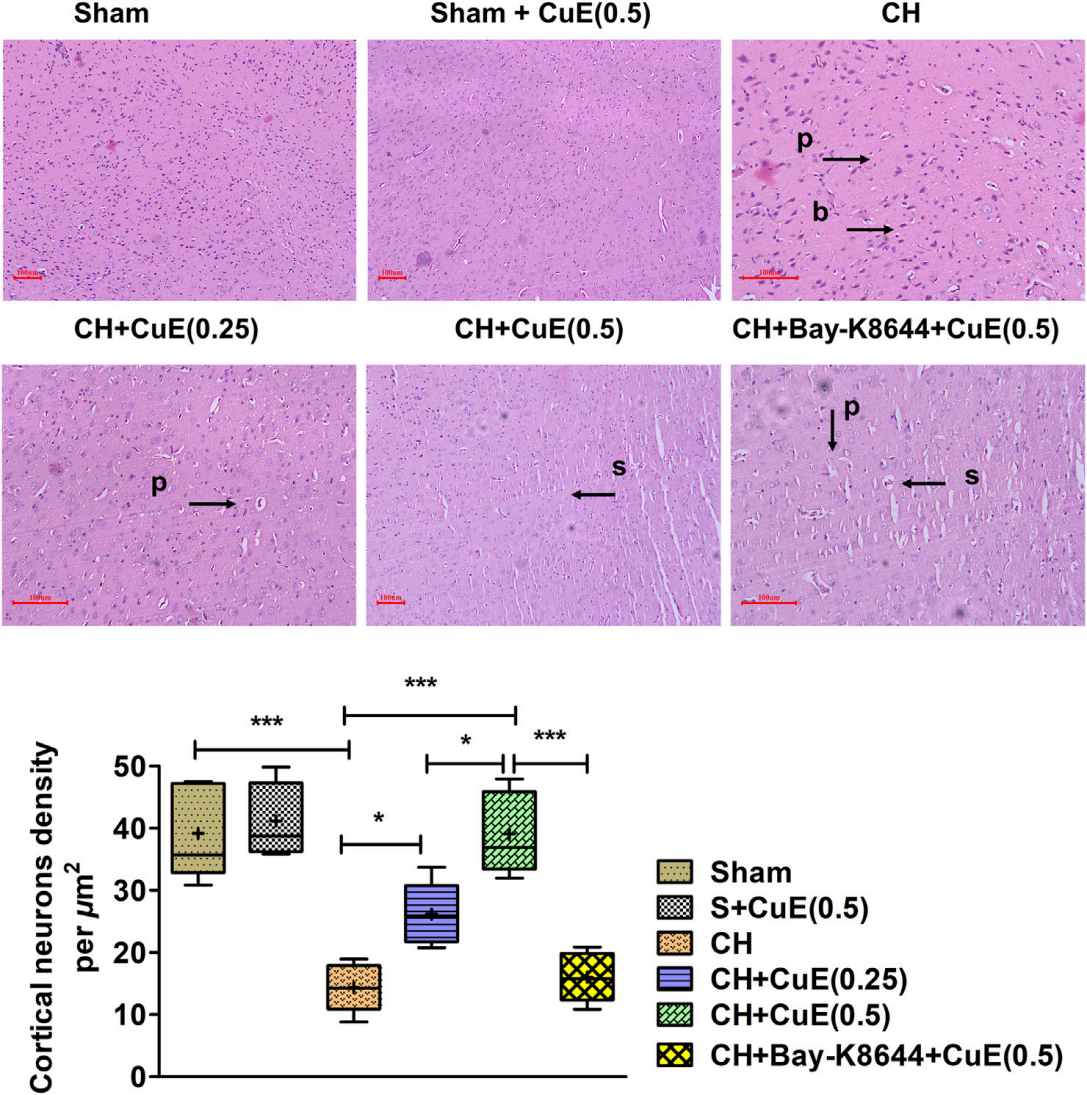
CuE, 0.25 and 0.5 mg/kg, *p.o.*, attenuated CH triggered neurodegenerative changes in the cortical regions (*n* = 5) (H & E stain, × 40, scale 100 µm). Pyknosis (p), bulging of plasma membrane (b), and swelling (s) were observed. Histomorphometric determinations indicate cortical neuron density per µm^2^. Box and whisker plots (Tukey) show mean (+), median (bold horizontal line), quartiles (box), and total range (whisker) (*n* = 5). ^*^ *p* < .05, ^**^ *p* < .01, ^***^ *p* < .001

In histopathological examination using H&E stain, rats subjected to permanent BCCAo exhibited neurodegenerative deviations highlighted by blebbing of the plasma membrane (b), swelling (s), and pyknosis (p) in the cortex areas of the brain. Sham rats displayed no signs of neurodegeneration. Administration of rats with CuE (0.25 and 0.5 mg/kg) attenuated CH-induced neuropathological changes in plasma membrane and genetic material (Figure 8). Bay-K8644 was found to enhance the neurodegenerative signs in the brain of rats subjected to CuE (0.5 mg/kg) treatment and CH. CH significantly reduced (*p* < .001) the cortical neuron density relative to sham. Viable neuron density was significantly increased by CuE (0.25 and 0.5 mg/kg) in the cortex (*p* < .05; *p* < .001) [F_(5,29)_ = 23.21, *p* < .001] that were subjected to CH. CuE (0.5 mg/kg) caused substantial increase in cortical neuron density (*p* < .05) relative to CuE (0.25 mg/kg) in *p*-BCCAo operated rats. Bay-K8644 (Ca^2+^ agonist) significantly attenuated CuE (0.5 mg/kg) induced protection of cortical neurons (*p* < .001) relative to CuE (0.5 mg/kg) in rats subjected to CH.

## Discussion

Several risk factors (e.g., age, cardiovascular disorders, metabolic disorders) and mechanisms related to vascular and hemodynamic alterations underlie the pathogenesis of CH and related disorders, such as dementia (Dong et al., 2018). Uninterrupted CBF is indispensable in the brain owing to glucose dependence, high metabolic rate, and energy recoupment *via* mitochondrial aerobic respiration (Duncombe et al., 2017). An inequality in the demand/supply relation of nutrients and oxygen to the brain owing to hemodynamic turbulences unfavorably affects the mitochondrial electron transport chain, oxidative phosphorylation, and cellular energy-homeostasis that accentuates free radicals, glutamate activity, ionic imbalance, recruitment of inflammatory biomolecules, amyloid-*β* burden, and tauopathy in the brain. Subsequently, electrical inactivity, edema, microangiopathy, and vascular resistance unite to cause irrevocable damage to the brain leading to extensive neurobehavioral discrepancies (Dong et al., 2018). Findings from prior studies suggest that cucurbitacins, particularly CuE, modulate diverse molecular targets and pathways that may protect against hypoperfusion states (Murtaza et al., 2017; Song et al., 2018; Xie et al., 2020). Although a few studies indicate promising effects of CuE in neurodegenerative disorders (Arel-Dubeau et al., 2014), nevertheless, the potential of CuE against perfusion defects has received very little emphasis so far.

Free radicals instigate endothelial damage and infiltration of leucocytes (neutrophils) *via* cellular lipid and protein modifications (Saxena et al., 2015). Free radicals readily react with polyunsaturated fatty acids that generate extremely reactive aldehydes (e.g., malondialdehyde, 4-hydroxy 2-nonenal, acrolein), which further modify proteins (Schiff base or Michael addition) and lipoproteins (e.g., low-density lipoproteins) (Chen et al., 2011). Malondialdehydes defunctionalize the autophagic process, form toxic adducts such as advanced lipid peroxidation end products (ALEs) (e.g., malondialdehyde-methylglyoxal), and react with genetic material (Liu et al., 2017). Under the hypoperfusion state, activation of iNOS culminates in nitrosative stress that leads to protein carbonylation and nitrosylation by peroxynitrites. Excess nitric oxide can cause irreversible cell injury, necrosis, and microvascular abnormalities by attenuating complex I and II activity in the mitochondrial electron transport chain, damage to DNA, and instigation of poly(ADP-ribose) polymerase (Daulatzai, 2017). In this study, CH caused increases in brain lipid peroxidation and DNA damage marked by increase in TBARS and 8-OHdG, which were substantially reduced by CuE post-treatment in rats. Hyperactivation of iNOS resulted in a disproportionate rise in protein carbonyls in rat brains exposed to CH. The present findings indicate that CuE treatment for 28 days daily alleviated the accumulation of protein carbonyls in the brain by decreasing the iNOS hyperactivity in rats against CH. Although antioxidants present in the brain scavenge the free radicals and detoxify dysfunctional proteins, however, under extreme oxidative stress, depletion of antioxidants severely hampers the defense mechanisms against CH. Experiments on transgenic animal models indicate that endogenous antioxidants, such as SOD and catalase, protect the brain against CH (Warner et al., 2004). Oral administration of CuE for 28 days increased the endogenous antioxidants (GSH, SOD, and catalase) in the brain of rats that were previously exposed to CH on day 1.

Hemodynamic aberrations damage the brain capillary network (e.g., BBB) by instigating unsolicited proteins (e.g., MPO and matrix metalloproteinases), cytokines (e.g., TNF-*α*), inflammatory transcription factor (e.g., NF-*κ*B), and reactive oxygen species (Wang et al., 2020), which, in turn, paves the way for invasion of neurotoxins, monocytes, neutrophils, and chemotactic factors in the brain parenchyma. TNF-*α* triggers caspase-associated apoptotic cell death and excitotoxic-nitric oxide pathway-mediated necrotic cell death. It augments transcription activity of NF-*κ*B that enhances expression of interleukins, matrix metalloproteinases, MPO, C-reactive protein, cell adhesion molecules, cyclo-oxygenase-2, and iNOS (Liu et al., 2017; Duncan et al., 2020). MPO in neutrophils, macrophages, and neurons promote oxidative stress and inflammation by catalyzing the production of hypochlorous acid (Chen et al., 2020). Activation of MMP-9 degrades the capillary vasculature (basal lamina) including BBB integrity (Vafadari et al., 2016). In the current study, CH increased TNF-*α* level, NF-*κ*B, MPO activity, and vascular injury biomarker MMP-9 in the brain of rats over a 28-day period. CuE post-treatment for 28 days significantly attenuated the TNF-*α* level, NF-*κ*B, MPO activity, and MMP-9 in the brain of rats subjected to CH. In this study, the biomarkers of necrotic cell death (LDH) and apoptotic cell death (caspase-3) were measured in the brain of rats. A significant increase in brain LDH activity and caspase-3 expression in rats subjected to CH indicated coparticipation of necrotic and apoptotic cell death mechanisms. The histopathological analysis well supported the biochemical findings. CH caused significant neurodegenerative changes marked by pyknosis, swelling, and blebbing of membranes in the cortical regions. However, CuE post-treatment for 28 days attenuated cell death and neurodegenerative changes in the CH rat model. Forebrain regions (e.g., cerebrum particularly temporal and parietal cortices) form the major seat of cognitive functions and processing of inputs and evidence indicate that these regions are most adversely affected during CH (Farkas et al., 2007). Histomorphometry analysis showed that CuE protected the neurons against CH in the cortical portion of the brain of rats marked by a substantial higher number of viable neurons. Bay-K8644 attenuated the prosurvival function of CuE in CH rat model.

Acetylcholine regulates cognitive functions by modulating synaptic plasticity, long-term potentiation, acquisition, encoding, consolidation, reconsolidation, extinction, and retrieval of memory (Resende and Adhikari, 2009). Acetylcholine plays an important part in regulating biological rhythms (e.g., wakefulness, sleep), stress response, inflammatory response, and vascular tone (Jin et al., 2015). In line with earlier findings (Sun et al., 2020), in this study, CH increased the AChE activity that might adversely affect the acetylcholine levels in the brain. Following the energy depletion and ionic imbalance, accumulation of glutamate in synapse depolarizes the postsynaptic membrane, triggers Ca^2+^ influx and nitric oxide release that establishes a vicious cycle of Ca^2+^-dependent catabolic (proteolytic) mechanisms (Belov Kirdajova et al., 2020). These excitotoxic pathways perpetuate inflammatory and oxidative mechanisms during CH. Recently, GABA is proposed to counter the glutamatergic excitatory drive in the brain. GABA can impart neuroprotective, antioxidant, antiapoptotic, and anti-inflammatory effects through improvement in CBF, glucose utilization, and energy production (Chen et al., 2019; Ngo and Vo, 2019). Because hyperpolarization of neurons decreases the metabolic rate, free radicals, and inflammatory cascade, analogous to hypothermia (Neumann et al., 2013; Lee et al., 2018), GABA refereed hyperpolarization of neurons is a documented neuroprotective tactic used in brain ailments involving excitotoxic pathogenesis. In this study, CuE post-treatment in rats attenuated the brain AChE activity and glutamate level and enhanced the GABA levels in the CH model.

In the current study, neurological scores, sensorimotor performance, and memory functions were evaluated in all the animals. Results displayed that CH severely hampered the neurological and motor functions over a 4-week period. CuE post-treatment improved the neurological and sensorimotor functions evident by a decline in mNSS and increase in latency to fall from the rotating rod, respectively, in the CH rat model. Earlier findings also substantiate CH-triggered decrease in neurological and motor performance and damage to memory skills in animals (Yanpallewar et al., 2005; Bhuvanendran et al., 2019). In the present study, oral administration of CuE significantly increased DI and STL, which highlighted enhancement in the memory performance of rats exposed to CH on day 1. Furthermore, we detected a dose-dependent betterment of biochemical parameters and neurobehavioral activity in rats by CuE. Administration of CuE (0.5 mg/kg) exhibited a substantial improvement in the parameters of oxidative stress, inflammation, cell death, and neurotransmitters in comparison to CuE (0.25 mg/kg) in rats against CH. Commensurate with biochemical findings, CuE (0.5 mg/kg) enhanced the cognitive functions relative to CuE (0.25 mg/kg) in rats against CH.

**FIGURE 9:**
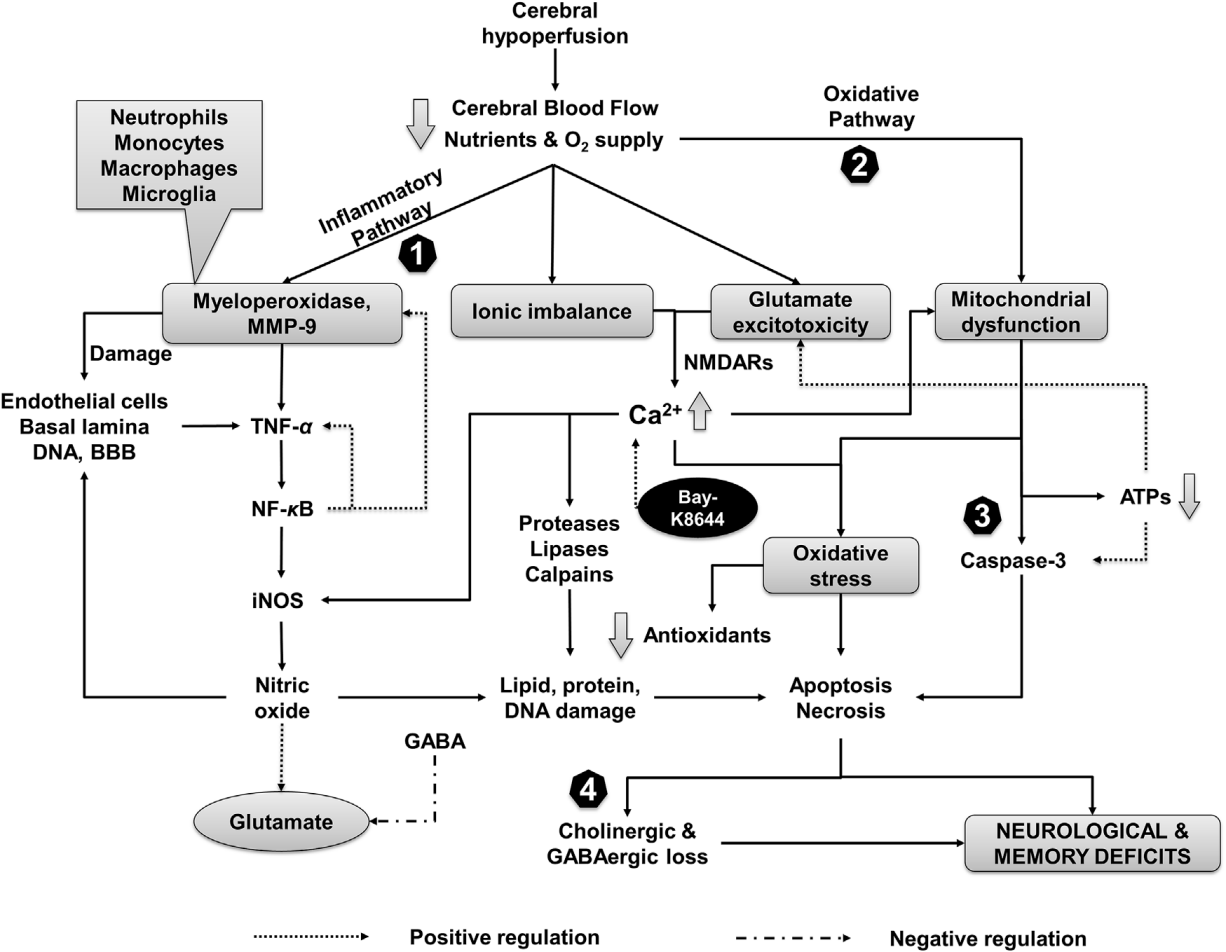
Events associated with CH injury and putative neuroprotective mechanism of CuE. Inflammation, oxidative stress, and ion influx are the primary events associated with reduced CBF followed by derangement of neurotransmitters. CH recruit MMP-9 and myeloperoxidase leading to sequential activation of TNF-*α*, NF-*κ*B, and iNOS. CH-induced formation of reactive oxygen species and mitochondrial dysfunction leads to decline of endogenous antioxidants and damage to biomolecules (e.g., lipids, proteins, genetic material). A decrease in adenosine triphosphates (ATPs) further potentiates the glutamate toxicity and activates apoptotic and necrotic pathways of cell death through caspases in the brain. Ionic imbalance and glutamate excess increase the intracellular calcium (Ca^2+^) levels that aggravates oxidative stress and activate proteases, lipases (e.g., phospholipase A_2_), and Ca^2+^ dependent calpains. Reduction in GABAergic transmission in response to CH paves the way for undeterred glutamate excitotoxicity. CuE attenuates inflammatory cascade (1) and free radicals (2) that protect brain against proteolytic damage (3). Improvement in GABA levels by CuE antagonizes the CH triggered glutamate toxicity (4) and enhances the cholinergic function in the brain against CH leading to neuroprotective effects and improvement in cognitive functions. Bay-K8644 (Ca^2+^ agonist) can enhance the Ca^2+^-induced catastrophe and antagonize the brain protective effects of CuE in CH rat model.

Interestingly the current findings highlight that Bay-K8644 (Ca^2+^ agonist) attenuates the antioxidative and anti-inflammatory effects of CuE (0.5 mg/kg) in a rat model of CH that promoted brain damage evident by an increase in caspase-3. Glutamate induced *N*-methyl D-aspartate receptor hyperactivation is known to enhance the cytoplasmic Ca^2+^ levels that trigger catastrophic cell death pathways. Furthermore, Ca^2+^-induced excessive nitric oxide biogenesis perpetuates this vicious cycle, resulting in profound brain damage. In the present study, administration of Bay-K8644 revealed a pathogenic increase in iNOS, protein carbonyls, and glutamate levels in the rat CH model that substantiated involvement of Ca^2+^ pathways in cerebroprotective effects of CuE in the current prototype. Bay-K8644 significantly attenuated the CuE induced decrease in AChE activity and rise in GABA content in rats subjected to CH. Behavioral data also reflected a decrease in neurological, sensorimotor, and cognitive performance of rats treated with Bay-K8644. Taken together, it might be possible that CuE exerted the antioxidative, anti-inflammatory, neuroprotective, and memory-enhancing effects *via* suppression of Ca^2+^-linked pathways in the CH state (Figure 9).

## Conclusion

The findings of the present study suggest that CuE possess therapeutic worth against CH. CuE mitigated neurobehavioral discrepancies against CH by antioxidant, anti-inflammatory, and neuroprotective effects. CuE can ameliorate brain cholinergic neurotransmission by declining AChE activity and has the potential to alleviate glutamate excitatory drive *via* GABAergic activity that may resurrect neurobehavioral functions in hypoperfusion states. The current research findings specify that CuE has potential against CH-associated cerebral disorders.

## Data Availability

The raw data supporting the conclusion of this article will be made available by the authors, without undue reservation.
