# Corrigendum: The Mechanisms of Cucurbitacin E as a Neuroprotective and Memory-Enhancing Agent in a Cerebral Hypoperfusion Rat Model: Attenuation of Oxidative Stress, Inflammation, and Excitotoxicity

**DOI:** 10.3389/fphar.2021.844464

**Published:** 2022-01-21

**Authors:** Zhiyong Liu, Manish Kumar, Sushma Devi, Atul Kabra

**Affiliations:** ^1^ Henan University of Traditional Chinese Medicine, Zhengzhou, China; ^2^ Chitkara College of Pharmacy, Chitkara University, Punjab, India; ^3^ Department of Pharmacy, Guru Nanak Institute of Technology, Ambala, India; ^4^ University Institute of Pharma Sciences, Chandigarh University, Mohali, India

**Keywords:** cerebral hypoperfusion, cucurbitacin E, memory, GABA, bay-K8644, caspase-3, inflammation, working memory

There is an error in the **Funding statement**. The correct Name for the Funder is “Special Research Project of Traditional Chinese Medicine in Henan Province (Grant No. 20-21ZY1024)”.

The authors apologize for this error and state that this does not change the scientific conclusions of the article in any way. The original article has been updated.

